# Supplementary material for: Comparative Analysis of Mafura Butter Oils from Trichilia emetica and Trichilia dregeana Extracted by Screw-Press from Seeds Collected in KwaZulu-Natal Province of South Africa
Source: Plants (Basel). 2025 Oct 4;14(19):3071. doi: 10.3390/plants14193071 (PMC12526083; doi:10.3390/plants14193071)
Supplement: Supplementary file 1 [file plants-14-03071-s001.zip › plants-3847076-Supplementary S1.pdf]

Supplementary S1. Details of materials and methods used for physicochemical characterization, tocols and fatty acid profile analyses of oil extracted by screw press from *Trichilia emetica* and *Trichilia dregeana*.

1. Peroxide Value

The PV measurement was performed according to AOCS Cd 8-53 [45] and expressed as meq/kg of fat. 5 g of the oil sample was dissolved into 30 ml of acetic acid: chloroform (Merck) (3:2) in a flask (AOCS Cd 8-53). The flask was swirled before the addition of saturated potassium iodide. The mixture was subjected to an excess of iodide via a saturated solution of potassium iodide (0.5 ml). The solution was swirled again for 1 min. The peroxides oxidized the iodide to iodine, and the iodine was titrated to a colorimetric endpoint (blue colour disappeared) using 0.01 N sodium thiosulfate ($Na_2S_2O_3$) solution (standardized using potassium dichromate and potassium iodide, Merck) with potato starch as an indicator. The amount of produced iodine was directly proportional to PV.

2. Free Fatty acid Value

The free fatty acid value was performed according to AOCS Method Cd 3d-53 [45]. 5 g of the sample was dissolved in 30ml neutralised isopropyl alcohol and toluene (equal parts by volume) containing 1% phenolphthalein indicator in ethanol (Merck). After the sample was completely dissolved, the test portion was swirled while titrating with 0.1M Reagent grade Potassium Hydroxide (Merck) 0.1 M to the first permanent pink colour of the same intensity as that of the neutralized solvent, before the latter was added to the test portion. The volume was recorded after the colour persisted for 30s.

3. *p*-Anisidine value

p-Anisidine value (meq/kg) was determined by a spectrophotometric method according to AOCS Cd 18-90 [45]. 0.5 of the oil sample was weighed into a 25-ml volumetric flask and diluted to volume with 5–10 ml spectrophotometric grade isooctane (Merck). Then, the absorbance of the sample was measured at 350 nm as a blank using an ultraviolet/visible spectrophotometer (Peak C-7200.). Next, 5 ml of the solution was pipetted into the test tube and 5 ml of the isooctane solvent into the second test tube. One mL of the *p*-anisidine solution was poured into each of the test tubes. After 10 min, the absorbance of the first test tube was measured at 350 nm using the second test tube as a reference. The *p*-AV was calculated using the formula given in the standard method (AOCS Cd 18-90). The formula used in the AOCS Cd 18-90 method for calculating the p-Anisidine Value (p-AV) is:

$$\text{p-Anisidine Value (p-AV)} = \left(25 \times \frac{1.2 \times A_2 - A_1}{W}\right)$$

Where:

- **A_1** = Absorbance of the fat/oil solution at 350 nm before reaction with p-anisidine.
- **A_2** = Absorbance of the fat/oil solution at 350 nm after reaction with p-anisidine.
- **W** = Weight of the fat/oil sample (in grams).

This formula measures the p-Anisidine value, which indicates the amount of aldehydes, particularly 2-alkenals, formed during the oxidation of fats and oils. The p-AV is used to assess the oxidative deterioration of oils.

4. Saponification Value

The test sample was filtered through dry filter paper to remove any impurities and moisture. 4.5 g of the test sample was weighed into an Erlen Meyer flask. 50ml of the alcoholic KOH (40g of potassium hydroxide in 1 litre of the distilled alcohol) (Merck) was added to the tests sample, connected to a condenser and boiled for one hour. After the flask and condenser had cooled somewhat, but not sufficiently to form a gel, the inside of the condenser was washed with a small quantity of distilled water. The condenser was disconnected, 1ml of 1% phenolphthalein indicator was added and the mixture was titrated with 0.5 M HCl until the pink colour just disappeared. The saponification value was measured by the formula given in the Standard method ACOS Cd 3-25 [45].

The saponification value was calculated using the following formula given in the Standard method ACOS Cd 3-25.

$$\text{Saponification Value (SV)} = \left(\frac{(B - S) \times N \times 56.1}{W}\right)$$

Where:

- **B** = Volume of hydrochloric acid (HCl) or sulfuric acid (H_2SO_4) used for titration of the blank (in mL).
- **S** = Volume of hydrochloric acid (HCl) or sulfuric acid (H_2SO_4) used for titration of the sample (in mL).
- **N** = Normality of the acid solution (in mol/L).
- **56.1** = Molar mass of potassium hydroxide (KOH), in g/mol.
- **W** = Weight of the sample (in grams).

5. Refractive Index

The Refractive Index was determined using and Atotago PAL-RI Refractometer. A zero reading was obtained using distilled water at 20°C and the measurement of the sample was also taken at 20°C.

6. Relative Density

The density was determined using a Guy-Lussac Pycnometer. The empty pycnometer was weighed on a 4 decimal balance (Bell M5-M214-Ai), where after it was filled with water at 20°C and weighed. The dried pycnometer was then filled with the sample at 20°C and weighed. The density of the oil and water was calculated where after the relative density of the oil was determined by determining the ration of the density of the oil to the ratio of the density of the water.

7. Carotenoid Content

The carotenoid content was determined by using Malasian Palm Oil Board method MPOB-P2.6.2004 [45]. The carotene content was determined by measuring the absorbance at 446nm of the oil sample dissolved in iso-octane. Carotenoids in most oils are mainly composed of beta-carotene. The result is given in assimilating the total carotene content to beta-carotene. 0.15 g of a completely homogenous and filtered sample was weighed, to the nearest 0.1 mg, into a 25 mL volumetric flask and dissolved in Isooctane (Merck, spectrophotometric grade). The absorbance was read at 446 nm, and the carotenoid content was measured by the following formula given in the MPOB-P2.6.2004.

$$\text{Carotenoid content (ppm)} = \left(\frac{\text{Absorbance at 446 nm} \times \text{Volume of sample (ml)} \times 10^{6}}{\text{Weight of sample (g)} \times 2592} \right)$$

Where:

- *Absorbance at 446 nm*: The absorbance reading of the sample at 446 nm.
- *Volume of sample (ml):* The volume of the sample used for the measurement.
- *Weight of sample (g):* The weight of the sample used.
- **2592**: A constant derived from the extinction coefficient for carotenoids in palm oil.

8. Iodine Value

Iodine values were calculated using method AOCS Cd 1c-85 [45] according to the potential number of iodine atoms added to each fatty acid or triglyceride.

9. Unsaponifiable material

Unsaponifiable matter was determined according to AOCS method Ca-6a-40 [45]. 5g of well mixed sample was weighed into an Erlenmeyer and saponified under reflux for 1 hr with 30 ml of 95% alcohol ethyl alcohol (Merck) and 5 ml of 50% KOH solution (Merck). The saponified mixture was transferred to the extraction cylinder and washed six times with petroleum benzine (Merck). The combined extracts were washed in the separatory funnel using ethanol in distilled water containing phenolphthalein, shaking vigorously, and drawing off the aqueous alcohol layer after each extraction until the wash solution no longer gave a pink colour. The solvent was transferred to a flask, and the solvent was evaporated and the unsaponifiable residue was determined gravimetrically. The weight of the residue was corrected using titration to determine the fatty acid content.

10. Fatty acid profile

The determination of the fatty acid composition procedure was based on AOCS method Ce2-66 [46] by preparing methyl esters which are separated and determined by Gas Chromatography using flame ionization detection. A 14% BF_3 (Sigma-Aldrich) reagent was used for derivatisation and transesterification with 0.5M NaOH in methanol (Sigma-Aldrich). The derivatized sample was taken up in 2 mL of heptane (Merck) and 1ul of the sample was injected onto a Restek Rt-2560 100m column, 0.25mmID and 0.20um film thickness (Restek). The oven program started at 100°C for 4 min and then increased at 3°C/min to 240°C where after it was held for 10 minutes. The injector was set at 225°C and the detector was set at 250°C. Hydrogen was used as carrier gas (Afrox, South Africa). The fatty acids were expressed as g/100g total fatty acids. An external fatty acid methyl ester mixture (Supelco 37 Component FAME mix 10 000 ug/ml in CH_2CL_2) was used to identify the fatty acids.

11. Determination of tocols

11.1 Materials and Equipment

The determination of tocols (tocopherols and tocotrienols) was done according to the ISO methods ISO 9936 [46]. HPLC grade n-hexane and 1,4-dioxane were purchased from Sigma-Aldrich. The mobile phase for the HPLC consisted of a mixture of n-hexane and 1,4-dioxane

(96:4) which was prepared and sonicated prior to use. The alpha-tocopherol standard (97.70 %) was purchased from Sigma-Aldrich.

Tocopherols and tocotrienols were measured by normal phase HPLC using a Sykam S 5300 sample injector, Sykam S 1130 HPLC isocratic pump system and a Waters 2475 fluorescence detector with excitation at 295 nm and emission at 330 nm. Separation was obtained by injecting 20 µL of the solution onto a Luna 5 µm silica 100 Å LC column 250 x 4.6 mm and applying a flow rate of 1.5 ml/min

11.2 Procedure

A stock solution of alpha-tocopherol was prepared by dissolving 10 ± 1 mg alpha-tocopherol in HPLC grade n-hexane in a 50 mL volumetric flask (± 200 ppm). The true concentration of the solution was calculated after the maximum absorbance of the solution was determined at 292 nm, using an UV spectrometer (Peak 7200 UV/VIS). The stock solution was then diluted by adding 9 mL HPLC grade n-Hexane to 1 mL of the stock solution. Samples were prepared in subdued light by dissolving 0.25 ± 0.1 g of the sample into a 25 mL amber volumetric flask, using HPLC grade n-hexane. The solutions were filtered through a syringe PTFE 0.45 µm filter and transferred to amber reaction vials. The diluted alpha-tocopherol solution was injected (20 µL, in duplicate) and the areas of the alpha-tocopherol peaks were recorded and multiplied by 10 to obtain the true areas. Similarly, the samples were also injected in duplicate and the tocopherols and tocotrienols were identified. Alpha-tocopherol was used as the only reference for the calculation of the tocopherols and tocotrienols. The individual tocopherol and tocotrienol contents were calculated according to the following formular in as per the ISO 9936 method.

$$\text{Tocopherol or Tocotrienol content (mg/kg)} = \left(\frac{\text{Peak area of analyte} \times \text{Concentration of standard} \times \text{Volume of sample injection (mL)}}{\text{Peak area of standard} \times \text{Weight of sample (g)} \times \text{Dilution factor}}\right)$$

Where:

- **Peak area of analyte** is the area under the curve corresponding to the tocopherol or tocotrienol in the sample.
- **Concentration of standard** is the concentration of the tocopherol or tocotrienol standard used for calibration (usually in mg/mL).
- **Volume of sample injection (mL)** is the volume of the sample injected into the HPLC system.
- **Peak area of standard** is the area under the curve corresponding to the tocopherol or tocotrienol standard.
- **Weight of sample (g)** is the weight of the sample being analyzed.
- **Dilution factor** is any dilution made to the sample prior to analysis.
